# Graph Theoretic and Motif Analyses of the Hippocampal Neuron Type Potential Connectome

**DOI:** 10.1523/ENEURO.0205-16.2016

**Published:** 2016-11-18

**Authors:** Christopher L. Rees, Diek W. Wheeler, David J. Hamilton, Charise M. White, Alexander O. Komendantov, Giorgio A. Ascoli

**Affiliations:** Krasnow Institute for Advanced Study, George Mason University, Fairfax, VA 22030

**Keywords:** connectomics, graph theory, hippocampal neurons, motifs, network, neuroinformatics

## Abstract

We computed the potential connectivity map of all known neuron types in the rodent hippocampal formation by supplementing scantly available synaptic data with spatial distributions of axons and dendrites from the open-access knowledge base Hippocampome.org. The network that results from this endeavor, the broadest and most complete for a mammalian cortical region at the neuron-type level to date, contains more than 3200 connections among 122 neuron types across six subregions. Analyses of these data using graph theory metrics unveil the fundamental architectural principles of the hippocampal circuit. Globally, we identify a highly specialized topology minimizing communication cost; a modular structure underscoring the prominence of the trisynaptic loop; a core set of neuron types serving as information-processing hubs as well as a distinct group of particular antihub neurons; a nested, two-tier rich club managing much of the network traffic; and an innate resilience to random perturbations. At the local level, we uncover the basic building blocks, or connectivity patterns, that combine to produce complex global functionality, and we benchmark their utilization in the circuit relative to random networks. Taken together, these results provide a comprehensive connectivity profile of the hippocampus, yielding novel insights on its functional operations at the computationally crucial level of neuron types.

## Significance Statement

Brain connectomes are being constructed at two disjointed levels. Microscopically, the wiring of individual neurons is being accumulated into cumbersome synaptomes; macroscopically, region-to-region projectomes obscure important circuit details. Neuron types provide a fertile middle ground. Using the 122 hippocampal formation types from Hippocampome.org, we augmented sparse connectivity knowledge with morphological evidence to obtain a full potential connectome. Though this network contains >3200 connections that are not easily amenable to intuitive hypothesis generation and testing, such complexity can be tackled using graph theory analysis, whereby we investigate the relationship between the circuit’s connectivity properties and functions. As type-level data grows, the array of analyses detailed here can be extended to rapidly supplement our understanding of the computational operation of the hippocampus.

## Introduction

The rodent hippocampus encompasses millions of neurons (West et al., 1991; Hosseini-Sharifabad and Nyengaard, 2007; Bandeira et al., 2009; Fu et al., 2013), each synapsing with tens of thousands of others (Gulyás et al., 1999; Megías et al., 2001). Examination and quantitative analysis of anatomical connectivity constitute a critical step toward understanding the circuit function (Sporns et al., 2005).

Major community efforts such as the BRAIN Initiative (Insel et al., 2013) and the Human Brain Project (Markram et al., 2015) are currently attempting to reconstruct the entire synaptic connectivity of each individual neuron. These undertakings produce massive datasets, but their necessary focus on extremely contained anatomical domains cannot comprehensively reveal long-range circuit architecture (e.g., Mishchenko et al., 2010; Kasthuri et al., 2015). At the other extreme, approaches such as the Human Connectome Project (van Essen et al., 2013) and the Allen Mouse Brain Connectivity Atlas (Oh et al., 2014) use diffusion tensor imaging or anterograde/retrograde tractography to map brain-wide regional connectivity (see also Mitra, 2014; Zingg et al., 2014). However, the limited spatial resolution and lack of cellular specificity restrict the utility of these data to inform our understanding of neuronal computation.

In between these popular synaptome (DeFelipe, 2010) and projectome (Kasthuri and Lichtman, 2007) levels lies an arguably more immediately fertile neuron-type circuitry approach that intuitively harmonizes well with Cajal’s “neuron doctrine” (Shepherd, 1991). Although neurons are indeed unique cellular units, they may be readily grouped according to sets of properties that cluster along a continuum. Over the past 6 years, we mounted a massive literature search to catalog all known neuron types in the rodent hippocampal formation based on their main neurotransmitter, axonal-dendritic morphologies, somatic location, molecular expression, and electrophysiological parameters (Wheeler et al., 2015). All the properties (and underlying experimental evidence) of the resulting 122 neuron types are collated in a publicly available, highly curated knowledge base (Hippocampome.org) that is ripe for analysis along multiple dimensions.

Here, because knowledge about synaptic connectivity among the types is sparse, we fill the gaps by exploiting Peters' rule (Braitenberg and Schüz, 1991), which recognizes axon–dendrite juxtapositions among the types as potential connections. We then quantitatively examine the resulting complex network using graph theory (Bullmore and Sporns, 2009; Rubinov and Sporns, 2010; Wig et al., 2011; Binicewicz et al., 2015). Through a suite of analyses, we investigate global degree distribution, circuit modularity, rich club coefficients, absorption, and driftness, as well as local motif composition, to foster intuition on how the functionality of the hippocampus relates to its fundamental architectural properties (Sporns et al., 2000). We also present an interactive, online, open-source toolbox for exploring the potential neuron-type connectome in the rodent hippocampal formation.

## Materials and Methods

### Identification of neuron types

This work focuses on rodent (mouse and rat, of either sex) hippocampal formation, defined as the dentate gyrus (DG), CA3, CA2, CA1, subiculum, and entorhinal cortex (EC). Each of these subregions is divided in layers (e.g., CA3 oriens, pyramidale, lucidum, radiatum, and lacunosum-moleculare; or EC L1–L6) giving rise to a total of 26 anatomical parcels. Over a period of several years, we amassed information on hippocampal formation neuron types from the century-deep and information-rich body of literature. However, because neurons are often named on an *ad hoc* basis without full mappings to previous names and descriptors (Hamilton et al., 2016), author-provided names of types were treated warily. Instead, neuron types were identified chiefly based on their primary neurotransmitter (i.e., glutamate or GABA) and for having a unique binary pattern of axonal and dendritic presence or absence across the 26 parcels (Wheeler et al., 2015). In rare cases (e.g., fast-spiking/parvalbumin-positive and regular-spiking/cholecystokinin-positive basket cells, ivy and bistratified cells), aligned molecular marker and electrophysiological evidence was sufficiently different to support the creation of two distinct types out of neurons with the same morphological pattern and primary neurotransmitter. Type names were then selected, differentiated, combined, or created anew to minimize confusion with the existing literature and fully mapped to their synonyms (Hamilton et al., 2016). The complete set of terms, definitions, data, and supporting experimental evidence collectively underlying the identification of the resulting 122 hippocampal neuron types is publicly available in open access form at Hippocampome.org (RRID: SCR_009023). Table 1 provides a glossary of neuron types to facilitate identification in figures throughout this article.

**Table 1.** Neuron type glossary.

1	Granule
2	Hilar Ectopic granule*
3	Semilunar granule
4	Mossy
5	Mossy MOLDEN*
6	AIPRIM (Aspiny int w/proj. to SMi)
7	DG axo-axonic
8	DG basket
9	DG basket CCK^+^
10	HICAP
11	HIPP
12	HIPROM (Hilar int w/proj. to SMo)
13	MOCAP (molecular commissural-associational pathway related)*
14	MOLAX
15	MOPP
16	DG neurogliaform
17	Outer molecular layer*
18	Total molecular Layer
19	CA3 pyramidal
20	CA3c pyramidal
21	CA3 giant
22	CA3 granule
23	CA3 axo-axonic
24	CA3 horizontal axo-axonic*
25	CA3 basket
26	CA3 basket CCK^+^
27	CA3 BISTRATIFIED
28	CA3 interneuron-specific oriens*
29	CA3 interneuron-specific quad*
30	CA3 ivy
31	CA3 LMR-targeting
32	Lucidum LAX (lucidum axons)*
33	Lucidum ORAX (oriens axons)
34	Lucidum-radiatum*
35	Spiny lucidum
36	Mossy fiber-associated (MFA)
37	MFA ORDEN (oriens-dendrites)
38	CA3 O-LM
39	CA3 quadD-LM
40	CA3 radiatum*
41	CA3 R-LM
42	CA3 SO-SO (oriens-oriens)*
43	CA3 trilaminar
44	CA2 pyramidal
45	CA2 basket
46	CA2 wide-arbor basket
47	CA2 bistratified
48	CA2 SP-SR
49	CA1 pyramidal
50	Cajal-Retzius
51	CA1 radiatum giant
52	CA1 axo-axonic
53	CA1 horizontal axo-axonic
54	CA1 back-projection
55	CA1 basket
56	CA1 basket CCK^+^
57	CA1 horizontal basket
58	CA1 bistratified
59	CA1 int-specific LMO-O*
60	CA1 int-specific LM-R
61	CA1 int-specific LMR-R
62	CA1 int-specific O-R*
63	CA1 int-spec O-Target QuadD
64	CA1 int-specific R-O*
65	CA1 int-specific RO-O*
66	CA1 ivy
67	CA1 LMR
68	CA1 LMR projecting
69	CA1 neurogliaform
70	CA1 neurogliaform projecting
71	CA1 O-LM
72	CA1 recurrent O-LM
73	CA1 O-LMR
74	CA1 oriens/alveus
75	CA1 oriens-bistratified
76	CA1 O-bistrat projecting*
77	CA1 OR-LM*
78	CA1 perforant path-associated
79	CA1 perforant path quadD
80	CA1 quadrilaminar
81	CA1 radiatum
82	CA1 R-Recv apical-targeting*
83	Schaffer collateral-associated
84	SCR R-targeting*
85	CA1 SO-SO (oriens-oriens)
86	CA1 hipp-subiculum proj ENK^+^*
87	CA1 trilaminar
88	CA1 radial trilaminar
89	SUB EC-projecting pyramidal
90	SUB CA1-projecting pyramidal
91	SUB axo-axonic
92	LI-II multipolar-pyramidal
93	LI-II pyramidal-fan
94	MEC LII pyramidal-multiform
95	MEC LII oblique pyramidal*
96	MEC LII stellate
97	LII-III pyramidal-tripolar
98	LEC LIII multipolar principal*
99	MEC LIII multipolar principal*
100	LIII pyramidal
101	LEC LIII complex pyramidal*
102	MEC LIII complex pyramidal*
103	MEC LIII bipolar complex pyr
104	LIII pyramidal-stellate
105	LIII stellate
106	LIII-V bipolar pyramidal
107	LIV-V pyramidal-horizontal
108	LIV-VI deep multipolar
109	MEC LV multipolar-pyramidal
110	LV deep pyramidal
111	MEC LV pyramidal
112	MEC LV superficial pyramidal
113	MEC LV-VI Pyr-polymorph
114	LEC LVI multipolar-pyramidal*
115	LII axo-axonic
116	MEC LII basket
117	LII basket-multipolar int
118	LEC LIII multipolar int
119	MEC LIII multipolar int
120	MEC LIII superficial multiplr int
121	LIII pyramidal-looking int
122	MEC LIII superficial trilayer int

122 types ordered first by subregion, then by primary neurotransmitter, then alphabetically. Asterisks indicate types that are either not well known or contain relatively little molecular marker and electrophysiological evidence.

### Culling of known connectivity information

All 484 peer-reviewed literature references comprising version 1.0 of the Hippocampome.org knowledge base were mined in a first-pass attempt to determine which of the 14,884 (122^2^) directed pairs of neuron types are known to synapse or not to synapse. Information verified by various methods (e.g., electron microscopy, electrophysiological paired recordings) was annotated, and relevant quotes and figures were extracted. Future versions of Hippocampome.org will additionally examine sources that cite and that are cited by the original references, as well as search for specific peer-reviewed articles with neuron-type connectivity information.

### Computation of potential connectivity

**Figure 1.**
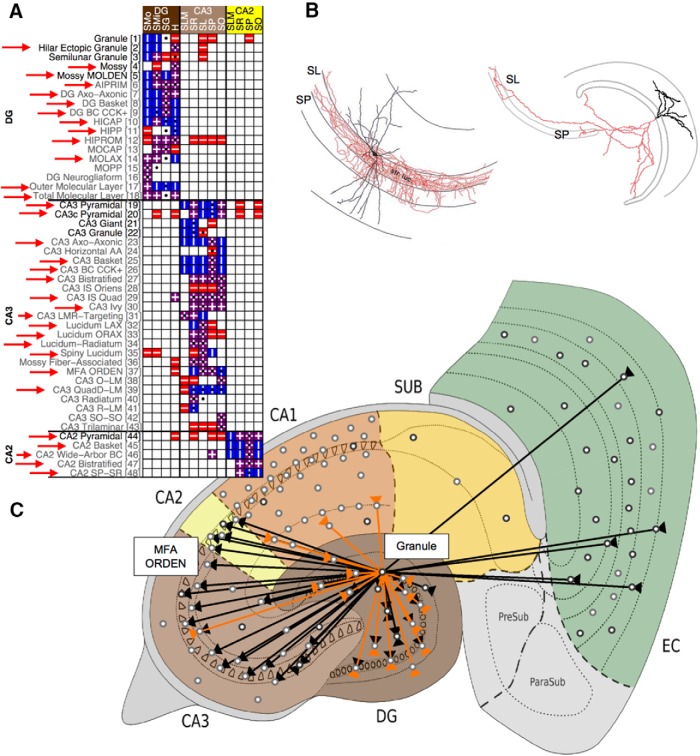
Potential connectivity of neuron types. ***A***, Partial matrix showing axonal and dendritic locations for selected DG, CA3, and CA2 types within certain parcels of the hippocampal formation (full matrix available online at Hippocampome.org/morphology). Bold, glutamatergic; gray, GABAergic; red boxes with horizontal lines, axons; blue boxes with vertical lines, dendrites; purple boxes with horizontal and vertical lines, both axons and dendrites; black circles, soma locations; red arrows, potential connections of granule cells. Parcel abbreviations for DG: SMo, outer stratum moleculare; SMi, inner stratum moleculare; SG, stratum granulosum; H, hilus; for CA3/CA2: SLM, stratum lacunosum-moleculare; SR, stratum radiatum; SL, stratum lucidum; SP, stratum pyramidale; SO, stratum oriens. ***B***, Representative illustration of the overlapping spatial distribution (indicative of potential connectivity) of granule cell axons (right) and MFA ORDEN cell dendrites (left) in CA3 SL and SP (for both neurons: axons in red; dendrites in black). Morphological reconstruction of the granule cell downloaded from NeuroMorpho.Org (Ascoli et al., 2007), with layers drawn in, from a tracing originally presented in Bausch et al. (2006). Permission to reprint the MFA ORDEN cell (Vida and Frotscher, 2000) granted by *Proceedings of the National Academy of Sciences of the United States of America* (Copyright 2000 National Academy of Sciences, U.S.A.). ***C***, Screenshot from the novel online toolbox (Hippocampome.org/connectivity) illustrating all information potentially received (arrows in) and sent (arrows out) by granule cells (black connections excitatory; orange inhibitory).

In the absence of literature evidence for known connections or nonconnections, information on potential connectivity between types was exploited to achieve a full hippocampal connectome (HC). The coexistence of the axons of one type with the dendrites of another within any hippocampal formation parcel indicates relative spatial proximity and a potential for synapsing. The rows in Figure 1*A* show a subset of neuron types and their defining axo-dendritic patterns. For example, the axons of granule cells are present in the DG hilus (H), CA3 stratum lucidum and stratum pyramidale, and CA2 stratum pyramidale. Therefore, any neuron type with dendrites in any one or more of these parcels, including mossy-fiber–associated oriens-dendrite (MFA ORDEN) cells (type 37 in Table 1), is a potential target of the granule cell axons (Fig. 1*B*). Further, types that do not have dendrites in those parcels are excluded as potential granule cell targets because of the lack of neurite overlap. This approach was extended to account for axo-somatic and axo-axonic connections of basket and chandelier cells, respectively. Mathematically, 26-dimensional binary vectors were used to encode the presence or absence across hippocampal parcels of the axon of each potential presynaptic type and of the dendrites (or soma or axonal initial segment) of each potential postsynaptic type. Potential connectivity was then calculated as the dot-product of these vectors: a nonzero result indicated a potential connection, whereas a zero-value dot-product indicated that connectivity was not possible between the types in question:

$$\text{Axons of type }\overset{\to }{A}=〈a_{1},a_{2},\ldots ,a_{26}〉;$$

$$\text{Dendrites of type }\overset{\to }{D}=〈d_{1},d_{2},\ldots ,d_{26}〉;$$

$$c=\overset{\to }{A}\;\;\cdot \overset{\to }{D}.$$

Many neuron types (101 of 122, not including granule cells) have axons and dendrites colocated within one or more parcels, indicating the potential for within-type connectivity; such self-connections are not necessarily indications of single-neuron autapses.

### Web-accessible resource for connectome visualization

A Java-based online toolbox was developed and deployed (Hippocampome.org/connectivity; click Launch or link to Potential Connectivity Map) to assist in the visualization and exploration of the HC. Glutamatergic (excitatory) and GABAergic (inhibitory) neuron types are represented as black and gray circles, respectively, and are placed randomly within the parcel (or along the parcel boundary) where their soma is most commonly located. Hovering over a type reveals its name; clicking on a type displays all the connections that may be received by its dendrites (lines with arrows in) or sent by its axons (arrows out). A snapshot of the toolbox, taken after the selection of granule cells, is shown in Fig. 1*C*. Toggles provide the ability to show or hide additional information, including connections made by the other (i.e. unselected) types and schematic illustrations of many of the major cell types and pathways in the hippocampal circuit.

### Graph theory analyses

The Brain Connectivity Toolbox (BCT; brain-connectivity-toolbox.net; Rubinov and Sporns, 2010) was used to compute many graph theory measures for the HC. In certain cases, the Matlab code was modified slightly to allow (or correct) for the possibility of self-connections of neuron types along the main diagonal of the connectivity matrix. In addition, some topological metrics were measured on the unweighted network, whereas others mandated connections weighted by the sign of the primary neurotransmitter of the presynaptic type: +1 for glutamatergic and –1 for GABAergic. To study the robustness of our results, we also examined a version of the network wherein connections of the most numerous principal cell (PC) types, namely DG granule cells, CA3 pyramidal cells, and CA1 pyramidal cells, were weighted as +10.

### Clustering coefficient, characteristic pathlength, and degree

Certain standard measures, including clustering coefficient (CC) and characteristic pathlength (CPL), are used to encapsulate the topology of the graph and are thus computed on a static, binary version of the network that disregards excitation and inhibition. Briefly, clustering coefficient is the fraction of connections among the immediate neighbors of a node (i.e., the set of neuron types that may receive information directly from that node) relative to the number of possible connections (Fagiolo, 2007). For example, granule cells have 33 immediate neighbors that are interconnected with 476 (of a possible 33^2^ = 1089) edges; CC_granule_ = 476/1089 = 0.437. This quantity, computed for each node, is then averaged over all neuron types to yield a single global value, CC_HC_.

Characteristic pathlength is defined as the mean of the shortest directed (i.e., axon to dendrite) path from a node to every other neuron type in the network. For example, granule cells and CA1 pyramidal cells are not in direct contact, so communication requires at least one intermediary; in fact, there are five two-step pathways (via types [19], [20], [44], [46], or [47]). Determining analogous distances from granule cells to the other types in the network and averaging gives CPL_granule_ = 2.11, meaning that they can send information anywhere in the network in an average of just over two steps. Then, averaging this quantity over all 122 types yields a single global value, CPL_HC_. Mathematically,

$$CPL_{HC}=\frac{1}{n}*\sum_{i=1}^{n}\left(\frac{1}{n}*\sum_{j=1}^{n}\text{shortest path }i,j\right),$$

where n is the number of nodes in the network, *i* is the set of presynaptic types, and *j* is the set of postsynaptic types. Neuron types that have axons and dendrites colocated within at least one parcel are self-connected and have a shortest pathlength to themselves of zero (e.g., the shortest path from CA1 pyramidal cells to CA1 pyramidal cells is 0); non–self-connected types require multistep paths to communicate with themselves (e.g., traveling from granule cells to granule cells requires two steps).

Node degree is the number of connections made by a node [out-degree (OD)], to a node [in-degree (ID)], or the sum of these quantities [total-degree (TD), also called degree centrality]. Again, granule cells have 33 immediate neighbors (OD_granule_ = 33), and they are immediate neighbors to 26 other types (ID_granule_ = 26); TD_granule_ = 33 + 26 = 59. Self-connected neuron types thus contribute two connections to their TD. A related measure, polarity, is defined as (ID – OD)/TD (Shih et al., 2015).

### Topology comparison analysis

For six well-known network types, we generated 1000 random networks identical in size to the HC and compared their CC and CPL. The two metrics were then combined to measure the overall (i.e., global and local) communication cost. Specifically, the cost was computed as follows:

$$\text{Communication cost}=-\log_{10}\left(CC\right)+\log_{10}\left(CPL\right).$$

For each network type, the resulting cost was linearly scaled so that the reference network (HC) was given unitary value.

The algorithms to produce the Erdös–Rényi (ER), lattice, ring, Watts–Strogatz (WS), Barabasi–Albert (BA), and Klemm–Eguílez (KE) networks were also implemented in Matlab (open-source code: github.com/Hippocampome-Org/GraphTheory) using published pseudocode (Prettejohn et al., 2011). Briefly, an ER network (Erdös and Rényi, 1960) is constrained only by its number of nodes and its connection density; we used HC network values of 122 and 21.7%. These graphs were constructed by considering all possible connections among the nodes and inserting them with probability equal to the connection density. A square lattice network, in contrast, is heavily constrained by the number of nodes and edges and the fact that each node must be connected to its *K* nearest neighbors (where *K* is the ratio between HC edges and nodes: 3236/122 = 26.5). A ring network, a one-dimensional string of nodes “bent” into circular form by joining the ends, is similarly constrained. Starting from a highly clustered ring graph, WS (Watts and Strogatz, 1998) networks were created by considering each connection for random rewiring with constant probability (*p*_rewiring_ = 0.4) to introduce long-distance (i.e., cross-network) edges. For BA scale-free networks (Barabasi and Albert, 1999), we started from an initial size of 10 fully connected nodes and serially attached the remaining 112 nodes to preexisting nodes chosen with probability proportional to their OD in the growing network. This preferential addition of new nodes to higher-degree nodes yields the desired power law distribution for the final network degree, with the vast majority of nodes having very small OD and a select few types having large OD. Finally, KE networks (Klemm and Eguíluz, 2002) are generated to obtain high CC and low CPL (like WS networks), along with a scale-free OD distribution (like BA networks). The algorithm is similar to that used for BA networks, but attachment of new nodes is preferentially biased toward high-degree, highly clustered “active” nodes (Prettejohn et al., 2011).

### Modularity

The HC modular, or community, structure was bared by computationally assigning neuron types into nonoverlapping groups to maximize within-community connectivity and minimize extramodular cabling. Community assignments are evaluated by a modularity score, Q, which quantifies the fraction of connections in a module relative to those expected by chance (Newman, 2004). Practically, we use BCT code based on a spectral algorithm that optimizes Q over possible HC divisions (Leicht and Newman, 2008). The algorithm was run 100 times, and the detected communities did not change.

### Rich club analysis

Rich club (RC) analysis used a modified version of BCT code to identify cores of nodes that are more highly connected to each other than expected by chance (Zhou and Mondragon, 2004; Colizza et al., 2006; McAuley et al., 2007; van den Heuvel and Sporns, 2011). First, a connectivity fraction (*C_f_*) is computed for each degree level *k* from 1 to the maximum TD in the network (i.e., 114, for CA3c pyramidal cells) as the proportion of edges that connect nodes of degree >*k* relative to the maximum number of edges that such nodes might share (Colizza et al., 2006). These *C_f_* values are then normalized relative to the average for a given *k* in a population of 1000 random networks synthetically generated to have fixed OD and ID distributions matching the HC. Raw *p* values were calculated at each *k* based on the *C_f_* percentile rank of HC within the population of 1000 random networks. Normalized *C_f_* values that were significantly greater than 1 over a range of *k*’s with *p* values smaller than 0.05 after false discovery rate multiple testing correction (Storey, 2002) were designated as members of RC tier I. The cutoff for inclusion in RC tier II was selected based on the relatively large *C_f_* increase in HC between *k* = 77 (*C_f_* = 0.630) and *k* = 78 (*C_f_* = 0.766).

### Absorption and driftness analyses

The shortest pathlengths between neuron types were again measured using BCT. The number of paths of length *y* between all pairs of types may be found simultaneously by multiplying the unweighted connectivity matrix, **M**, by itself *y* times (e.g., the matrix entries obtained by calculating **M** ^3^ are the number of paths of exactly three steps between types). Absorption and driftness values (Costa et al., 2011) were also computed in Matlab. The absorption metric simulates average random walks as a surrogate for dynamic activity in the network. In a given random walk from a starting neuron type to a destination, the walk progresses with equal probability to any of the connected types and continues until the target is reached. Averaging a large number of independent random walks mimics parallel propagation of activity over all possible paths connecting two neuron types. Driftness is calculated as the absorption value divided by the CPL for each pair of neuron types (Costa et al., 2011).

### Connectivity superpattern and pattern profiles

At a local level, we investigated the configurations of connectivity (or lack thereof) for all groupings of three neuron types. In a circuit with 122 elements chosen three at a time without regard to order, this equates to a total of 295,240 combinatorial relationships. In one analysis, we examined connectivity “superpatterns” without distinguishing excitatory and inhibitory connections (i.e., the network was considered directed but unweighted); in a second, we studied the directed and weighted network patterns. For the sake of interpretability, self-connections among types were not considered as differentiators in this analysis. Superpattern and pattern libraries and detection algorithms were built from scratch using Matlab (github.com/Hippocampome-Org/GraphTheory).

Excitability scores (ESs) quantify the net counterbalance of excitation versus inhibition occurring within a triad of neuron types. These scores are computed at each node in the pattern, then summed over the three nodes. If a node does not receive a connection from either of the two other nodes, its score is equal to its sign (+1 if excitatory node, –1 if inhibitory); this node is not amplified or dampened by the rest of the pattern. If a node receives a connection from one or both other nodes in the pattern, its score equals its sign multiplied by 1.1 for each incoming excitatory connection and by 0.9 for each incoming inhibitory connection. Explicit examples of this computation are included in Results.

Patterns may or may not have a unique ES. Thus, for each ES, we also quantified the relative prevalence within the detected modules to determine whether the underlying communities tended to use repeatedly certain sets of excitatory/inhibitory configurations. The relative importance of these interactions to a module is computed based on the number of times an ES appears within that module relative to the overall network.

### Detection of motifs and antimotifs

Analogously to the rich club analysis, counts of HC superpatterns and patterns were compared to a population of 1000 random networks to find those that were significantly over- or underutilized relative to expectancy, called motifs and antimotifs, respectively (Milo et al., 2002, 2004). These random networks were generated in parallel by selective edge swaps chosen stringently and conservatively so as to maintain the underlying spectrum of two-node (i.e., dimer) superpatterns and patterns. Specifically, the random networks preserved the HC number of excitatory (E) and inhibitory (I) nodes and connections; the number of E to E, E to I, I to E, and I to I connections; and the OD and ID of each node. Accordingly, each edge in the graph had a limited number of valid swap partners from which a suitable mate was randomly chosen. Fifty swapping passes were made over all edges to sufficiently scramble the original network.

The statistics used for the motif/anti-motif analysis were similar to those of the RC analysis: raw *p* values for each pattern were based on the percentile rank of the HC count within the random population. Patterns with percentile ranks >95 (meaning that the pattern appeared in the HC more than in 95% of the 1000 random networks) underwent multiple testing corrections to determine whether they constituted statistically significant motifs. Similarly, patterns with percentile ranks <5 underwent testing to identify antimotifs. Adjusted *p*-values were calculated with the step-down “min P” procedure (Westfall and Young, 1993), and patterns with corrected values <0.05 were deemed significant.

### Pairwise correlation analysis

Pairwise correlations were evaluated among 315 properties across the 88 neuron types in the DG, CA3, CA2, and CA1 subregions. In addition to connectivity properties detailed herein (degree, strength, polarity, and usage of superpatterns/patterns), we examined morphological features (e.g., somatic, axonal, and dendritic locations, as well as the projecting or local nature of axons), molecular markers (e.g., expression, or lack thereof, of various calcium-binding proteins, neuropeptides, or receptors), and assorted passive and spiking electrophysiological parameters (e.g., input resistance, fast or slow membrane time constants, action potential width). Neuron types from subiculum and EC were excluded from this analysis owing to the scarcity of available molecular and electrophysiological information. Direct and inverse relationships between properties were detected using 2-by-2 contingency matrices, and *p*-values were calculated with Barnard’s exact test (Lydersen et al., 2009).

## Results

In building the neuron type connectome of the hippocampal formation, we extracted information for 167 known connections and 68 nonconnections from the literature. For the remaining 14,649 type pairs (i.e., 122^2^ – 235 known connections or nonconnections), we calculated the spatially based potential connectivity, which excluded the possibility of connections for 11,580 pairs of neuron types. Consequently, 3069 potential connections were combined with 167 known connections to obtain the HC network explored here: a graph of 38 excitatory and 84 inhibitory neuron types (nodes) interlinked by 3236 edges (1216 excitatory and 2020 inhibitory; full connectivity data may be downloaded from Hippocampome.org/netlist).

### Highly specialized topology

**Figure 2.**
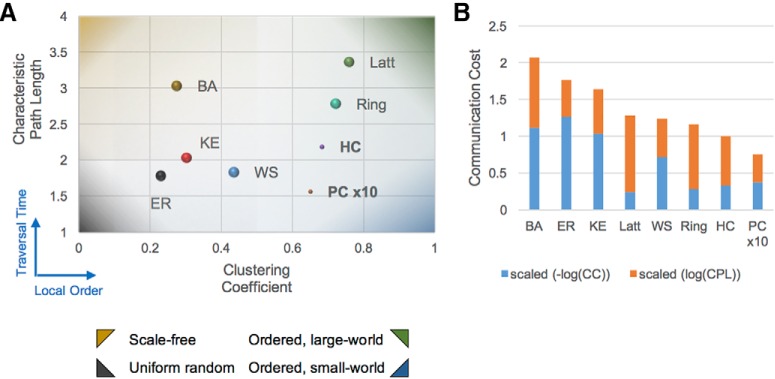
Comparison of the HC to well-known types of equivalently sized random networks. ***A***, Broad categorizations, indicated by background shading from the four corners, aid in grouping and analyzing network topology along two dimensions of interest: CC and CPL. Data points for six types of random networks are averaged from 1000-network datasets; standard deviations are illustrated by the diameter. ***B***, The combination of high CC and low CPL in the HC results in an optimally low overall communication cost in the network. BA, Barabasi–Albert; ER, Erdös–Rényi; KE, Klemm–Eguílez; Latt, square lattice; WS, Watts–Strogatz.

We first compared the HC clustering coefficient and characteristic pathlength to those of six identically sized, well-known network types (ER, BA, WS, KE, rings, and lattices). Because of the relatively small size of the graphs, CC and CPL showed little variance over the 1000 randomly generated variants of each network type (Fig. 2*A*). CC is indicative of the tendency of nodes to gather in tightly knit groups that may correspond to functional processing units, whereas CPL reveals the relative expanse of the network. Together, these metrics characterize network topology in terms of communication cost: large-world networks (Boccaletti et al., 2006) contain densely connected groupings of nearby nodes, but remote nodes are reachable only by paths with many steps (dark green background shading in Fig. 2*A*). At the opposite extreme, uniform random networks have low CC and CPL because of the arbitrary placement of their edges (dark gray shading). Scale-free networks (gold shading) and small-world networks (blue shading) represent two popular mixed cases, low CC/high CPL and high CC/low CPL, respectively. HC displays both high CC, analogous to rings and lattices, and low CPL comparable to ER random networks. This suggests that hippocampal neuron types rapidly combine information across short pathlengths into targeted areas, where specialized processing occurs within tightly interconnected circuits. In fact, not only is HC classifiable as a small-world network, but its combined global and local communication cost is lower than any of the other tested networks (Fig. 2*B*). Moreover, when PC connections were weighted 10 times more heavily than other edges, both CPL and the overall communication cost further decreased by 25%.

### Significant community structure

**Figure 3.**
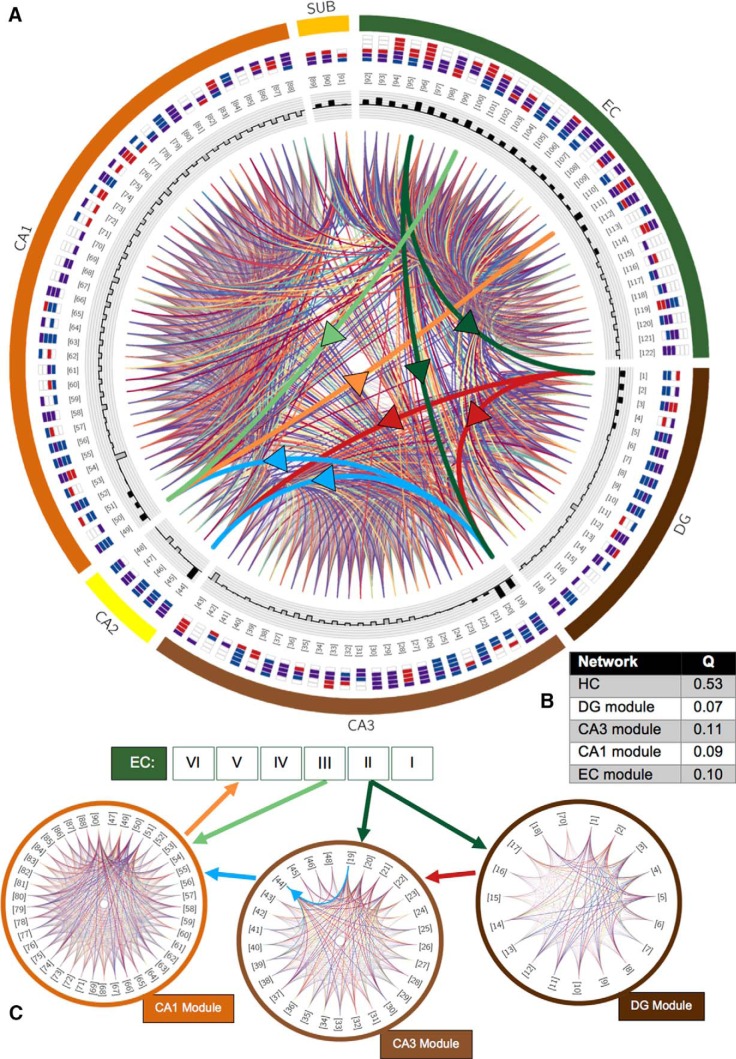
Modular structure of the potential hippocampal connectome. ***A***, Chord diagram of the potential connectivity among all 122 types (produced with Circos software: Krzywinski et al., 2009). Thick chords with arrows emphasize the trisynaptic loop (dark green, perforant pathway lines; light green, temporoammonic path; red, mossy fibers; blue, Schaffer collaterals; orange, projection from CA1 to EC layer V); other connections are colored randomly to optimize visibility. Types are identifiable by both numbers in brackets (names provided in Table 1) and axon-dendrite patterning within the subregion of their soma location (colored box convention and layer ordering from inside-out as in Fig. 1; layers for CA1: SLM, SR, SP, SO; for subiculum: stratum moleculare, SP, polymorphic layer; for EC: I–VI). Shaded bars in the innermost ring show the total number of (signed) connections made by that type; excitatory types have outward-facing black bars and inhibitory types inward-facing gray bars. ***B***, Modularity scores (Q) for the entire network and for the four detected modules. ***C***, The communities correspond closely to the DG (module connection density 75.9%), CA3 (59.3%), CA1 (64.6%), and EC (70.1%; not shown). Numbered types and colored arrows as in ***A***.

The organization of HC connectivity can be visually inspected on a circular graph (Fig. 3*A*). The innate community structure is identified by grouping the 122 neuron types to maximize intramodular wiring and minimize intermodular wiring. The modularity score Q measures the effectiveness of the resulting grouping, with values of Q ≈ 0 indicating randomness (i.e., the groupings are equally good or poor), and in practice, Q > 0.3 pointing to noteworthy community structure (Newman, 2004). The HC network is optimally subdivided into four modules with Q = 0.53. Connections between neuron types within one of these communities account for 81% (2622/3236) of all graph edges, and the average connection density of the four modules is 0.675, dwarfing the between-module connection density of 0.041 (614/3236). Interestingly, the communities do not themselves partition into smaller submodules, as the average Q score for each of the modules is 0.09 (Fig. 3*B*). Thus, the four detected communities are the major, high-level processing units of the network.

Even though axons of neuron types frequently cross subregion boundaries to form connections (i.e., 33 of 122 types project to different subregions from their soma location), the detected communities closely aligned with DG, CA3, CA1, and EC (the first three are shown in Fig. 3*C*), the most highly studied subregions of the hippocampal formation. These subregions are also the major players in the trisynaptic loop (TSL) relay (highlighted as thick, brightly colored chords in Fig. 3*A*). The DG module identified by this analysis contained all 18 DG types, along with one of the CA1 types that projects to DG (CA1 neurogliaform projecting). The CA3 module included all 25 types from CA3 and four of five types from less-researched CA2. The exception, CA2 bistratified cells, belonged to the CA1 module, along with the remaining 39 CA1 types and SUB CA1-projecting pyramidal cells. Finally, the EC module contained all 31 EC types and the other two subicular neuron types. Notably, this core modular structure was revealed even without differentially weighting the PC connections.

### Degree distribution and hubs

**Figure 4.**
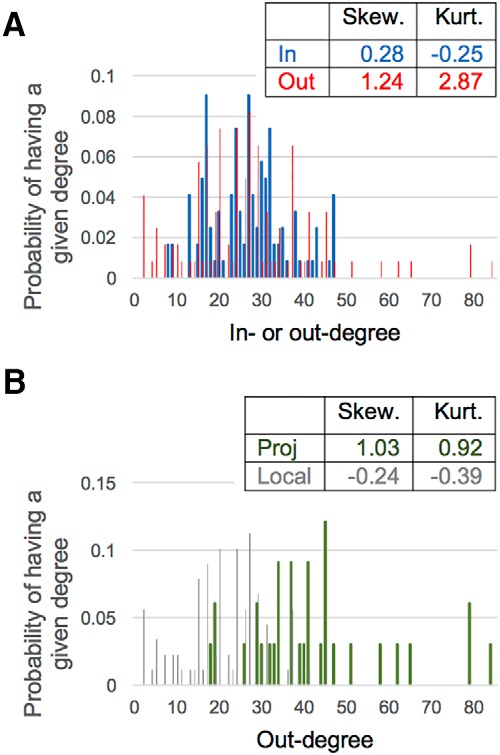
Breakdown of degree distribution to isolate neuron types with unusual connectivity. ***A***, Difference in the axonal and dendritic architecture is evident in the OD (red data series) and ID (blue) distributions. ***B***, The two OD tails are respectively attributable to highly connected hubs within the subset of neuron types that project to another subregion (green series; positive skewness) and to certain local neuron types with highly specific connectivity (gray; negative skewness).

The numbers of connections made and received by a neuron type respectively correspond to out- and in-degrees (Fig. 4*A*), and types with a relatively high TD may be generally considered network hubs (van den Heuvel and Sporns, 2013). The HC OD distribution (thin red columns) has both a more asymmetric and a more heavily tailed spread than the ID distribution (thick blue columns), as quantified by skewness and kurtosis values, respectively. Although both distributions have positive skewness, indicating a right-shifted distribution attributable to the presence of network hubs, the skewness of the OD distribution is more than four times larger. More strikingly, the OD kurtosis (2.87) denotes a much heavier tail than is found with the ID distribution, whose negative value close to zero indicates near-normal, if not thinner-than-normal, tails (Pearson, 1905; Westfall, 2014).

Altogether, this evidence points to an axonal architecture that is both anomalous and nonrandom in contrast to a relatively ordinary dendritic architecture. The peculiarity in the axonal distribution is accentuated by breaking down the data by neuron types that project to another subregion (Hippocampome.org/morphology) versus those with only local axons (Fig. 4*B*). The projecting types (thick green columns; *n* = 33) show a heavy, right tail versus the light, left tail of the local types (thin gray columns; *n* = 89). These tails indicate, separately, the presence of neuron types that serve as highly connected hubs and types that are decidedly particular in the connections they form.

The top and bottom neuron types by TD may be respectively considered global hubs and antihubs (Table 2). The list signals the importance of the CA3 module and highlights its central role in the TSL circuit: pyramidal cells from CA3, CA3c, and CA2 are three of the four most connected neuron types in the network. The other, CA1 back-projection cells, is an interneuron type in CA1 with axons that project upstream to CA3 (and DG), opposite to the TSL flow. Notably, granule cells are not a global hub based on pure topology (i.e., they are only the 43rd most connected neuron type), but they become the third most critical type in the 10× PC weighted network. Thus granule cells do not influence a large number of neuron types; rather, their importance in the HC is largely due to their relative abundance.

**Table 2.** Identification of hubs and antihubs with high and low TD, respectively.

Module	Neuron type	OD	ID	TD	Polarity
Global hubs					
CA3	CA3c pyramidal*	84	30	114	–0.47
CA1	CA1 back-projection^†^	79	25	104	–0.52
CA3	CA3 pyramidal*	65	33	98	–0.33
CA3	CA2 pyramidal*	79	9	88	–0.80
CA1	CA1 pyramidal*	41	46	87	0.06
CA1	CA1 quadrilaminar^†^	41	43	84	0.02
CA1	CA1 radial trilaminar^†^	37	47	84	0.12
CA1	CA1 LMR projecting^†^	45	35	80	–0.13
EC	MEC LV pyramidal*	51	27	78	–0.31
CA3	CA3 trilaminar^†^	62	13	75	–0.65
Global antihubs					
CA1	SUB axo-axonic^†^	2	15	17	0.76
CA3	CA3 horizontal AA^†^	2	13	15	0.73
CA3	CA2 basket^†^	5	9	14	0.29
CA3	CA2 SP-SR^†^	5	8	13	0.23
Provincial hubs					
DG	MOLAX^†^	17	32	49	0.31
CA3	CA3 bistratified^†^	26	28	54	0.04
CA3	CA3 ivy^†^	26	25	51	–0.02
CA1	CA1 oriens-bistratified^†^	36	24	60	–0.20

Polarities quantify the net in/out balance of information flow. *Excitatory; ^†^inhibitory.

Certain neuron types are global hubs because of a high OD even with a low ID (i.e., they have a dominant axonal architecture); others are more balanced. The top four global hubs have OD ≫ ID (i.e., a large, negative polarity) and constitute network divergence points. Global antihubs, with positive polarities, such as axo-axonic cells, basket cells, and interneuron-specific cells, indicate selected targets of information convergence. In contrast, provincial hubs, which by definition do not project outside of their module, are highly connected within the module and serve as critical traffic directors (van den Heuvel and Sporns, 2013). The top provincial hubs in the DG, CA3, and CA1 modules (bottom portion of Table 2) are again not restricted to a certain polarity. In DG, MOLAX cells tend to funnel information to specific points within the DG, but CA1 oriens-bistratified cells distribute information widely to 36 of 41 CA1-module types; CA3 bistratified and ivy cells have relatively balanced, neutral polarities.

### Rich and ultrarich clubs

**Figure 5.**
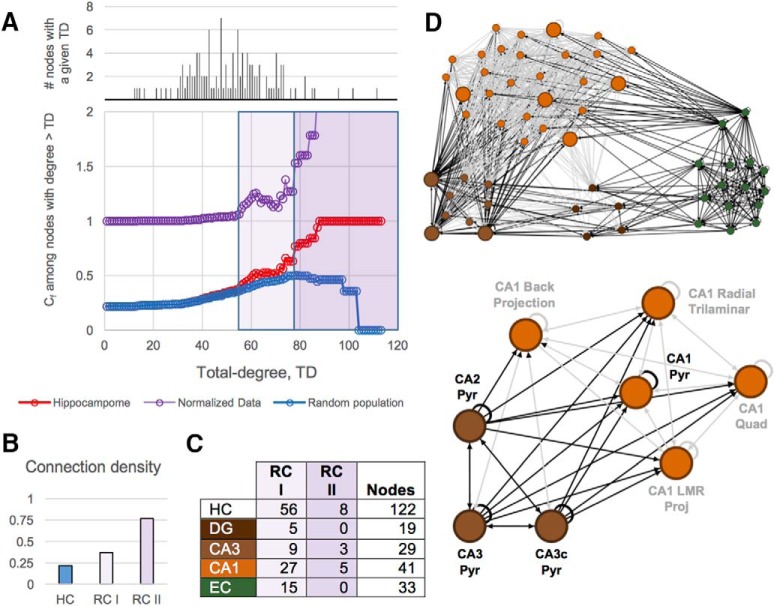
Nested rich clubs within the HC. ***A***, Top, distribution of nodes by TD; bottom, nodes with TD ≥55 are members of a densely interconnected rich club. Eight members of this club are also members of a second “ultra-rich” club level (dark purple shading; light purple shading indicates members of RC I but not RC II). ***B***, Connection densities of RC I and RC II are elevated compared with the rest of the network. ***C***, Modular analysis of each RC tier. ***D***, The 56 neuron types of RC I (top) and the subset constituting RC II (bottom).

Rich club analysis showed that the global hubs are significantly more connected among each other than could be expected by chance. In fact, all nodes with TD ≥ 55 (not just the top global hubs) have statistically higher interconnectivity than in equivalent random networks (Fig. 5*A*; normalized data > 1). Although nearly half (56 of 122; 45.9%) of the neuron types belong to this RC I (both light- and dark-purple shaded regions of Fig. 5*A*), the top eight hubs from Table 2 are also members of a tighter “richest of the rich” club within (RC II; dark-purple shading only), boasting the highest edge density of 76% (Fig. 5*B*). Members of RC I consist of types from all four modules (Fig. 5*C*), but a disproportionate number (42 of 56) come from CA1 and EC, as these modules are densely connected and have the most nodes. Interestingly, all RC II types are located in CA3 and CA1 (Fig. 5*D*).

### Robustness to random failures

**Figure 6.**
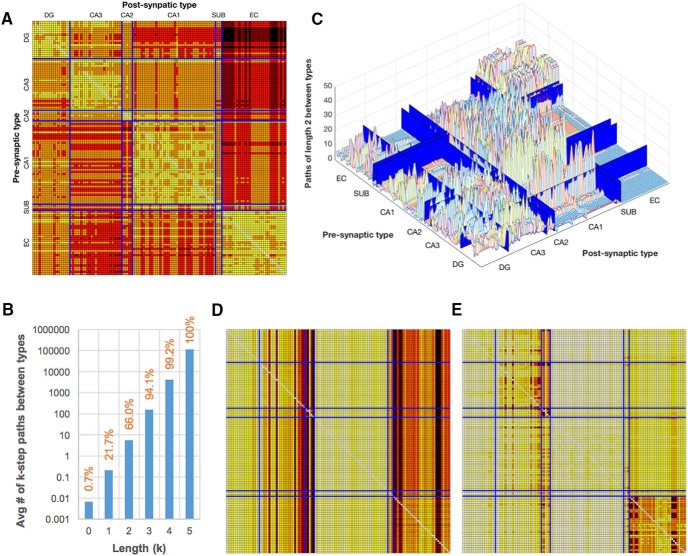
Alternate pathways between nodes afford resilience to the network. ***A***, Length of the shortest directed route between each pair of neuron types. Presynaptic types are in rows, postsynaptic types in columns; see Table 1 for type names and ordering. Color gradient key: yellow, direct connection; orange, red, and dark red, two, three, and four steps, respectively; black, highest pathlength (five steps). ***B***, Orange labels indicate the percentage of type pairs that can be bridged by a path of a given length, *k* (shown for *k* ≤ 5). In addition, at each *k*, blue columns show the average number of available conduits across all pairs of types. ***C***, For k = 2, peak height in a three-dimensional plot indicates the number of two-step paths between types. ***D***, Absorption measures the average length of all routes from (rows) and to (columns) other types. ***E***, Driftness is relatively low for most type pairs, pointing to the availability of multiple pathways between nodes that are similar in length to the shortest path. Color gradient for ***D*** and ***E*** as in ***A***.

Any two neuron types within a network can typically connect through multiple alternative pathways, providing redundancy for information flow. The maximum shortest pathway length in HC, five steps, was found only between certain DG and EC types, a route that requires upstream travel against TSL current (Fig. 6*A*). Approximately two thirds of all pairs of neuron types are connected by two steps or fewer, and nearly 95% by three (Fig. 6*B*, percentage labels). Moreover, increasing the length of possible paths by just a single step raises the number of available alternates by successive orders of magnitude (Fig. 6*B*, blue columns). For example, there are on average 5.81 available two-step pathways in HC between two neuron types. Although certain pairs have no such pathways (e.g., from DG to EC), others have many possible two-step routes at their disposal (Fig. 6*C*).

The absorption value for a pair of neuron types is the average length of all paths (the length of a “random walk”) between them (Fig. 6*D*). The overall average absorption of HC is 230.5. Whereas the out-absorption vectors (i.e., the rows in the matrix) are relatively similar across all nodes, the in-absorption vectors (columns) are highly specific to a given node. Therefore, neuron types are activated with just as much ease or difficulty from any part of the network, depending primarily on the dendritic architecture of the type and its close neighbors. CA2 and subiculum, being hard to reach with few connections arriving at a select few types, and EC, having overall unidirectional information flow through the TSL, have high in-absorptions. Driftness corresponds to the absorption normalized by the shortest pathlengths between the types (Fig. 6*E*). Intra-CA2 and intra-EC values are again high, but those for most other type pairs are low, indicating the existence of multiple pathways of similar scale to the shortest path. This feature suggests that the HC network could continue to operate at near-optimal levels after insult to random neuron types and connections. Notably, however, whereas the absorption values are unchanged when accounting for 10× PC weighting, the overall average driftness increases from 111.8 to 170.0. Thus, the “shortest” path between many types (i.e., through the PCs) is both unique and irreplaceable, and the PCs represent points of vulnerability.

### Characteristic connectivity superpattern profile

**Figure 7.**
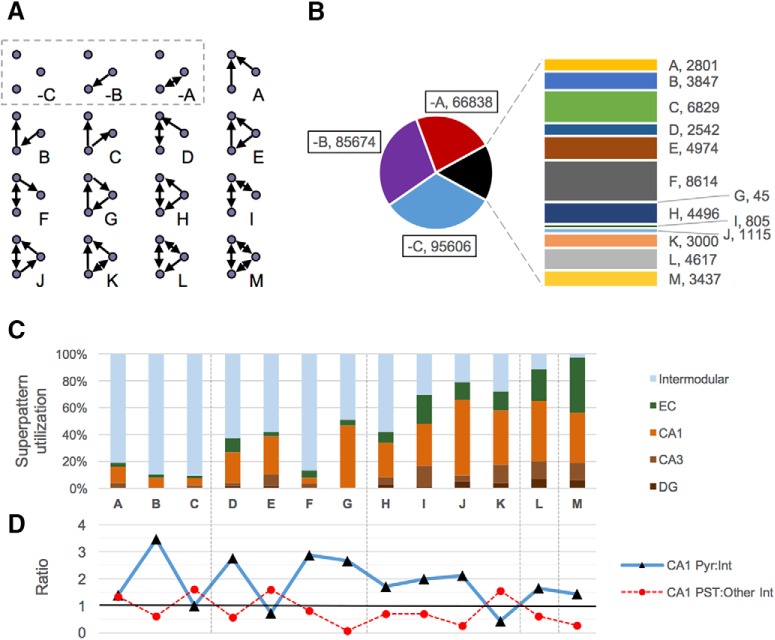
Superpatterns and HC usage. ***A***, Connectivity superpattern trimers are unweighted subgraphs of three nodes (disconnected superpatterns outlined in gray). ***B***, Counts of disconnected and connected superpatterns. ***C***, Percentage of connected superpatterns localized to a given module or found between modules. ***D***, Within CA1, superpattern usage also varies by cell type, as indicated by ratios of pyramidal cells to interneurons (blue line) and perisomatic interneurons to dendritic-targeting neurons (dotted red line).

To examine the local interactions of neuron types, we considered all 16 possible ways in which three unweighted nodes can interrelate: 13 in which all three nodes directly participate in at least one connection (Fig. 7*A*, graphs labeled A–M) and three with at least one node disconnected from the others (labeled –A, –B, and –C). The frequency of occurrence for these 16 building blocks constitutes the HC connectivity superpattern profile (Fig. 7*B*). An absolute majority of the 295,240 trimer occurrences in HC (248,118 or 84%) involve at least one disconnected type. Of the fully connected trimers, the superpattern consisting of a single uplinked mutual dyad (superpattern F; name modified from Milo et al., 2002) and the single input module (superpattern C; named after Zaslaver et al., 2004; Alon, 2007) represent nearly a collective one third (15,443 of 47,122; 32.8%). Most interactions take place intermodularly (Fig. 7*C*) involving projecting neuron types. In both F and C, a single node disperses information to two neuron types that do not interact with each other, with superpattern F receiving direct feedback from one partner and C containing no feedback from either partner. In contrast, superpattern I, a similar structure containing a chain of two mutual dyads in which both receivers of the dispersed signal provide direct feedback, is one of the least frequent. These observations point to a strong, net-unidirectional information flow between modules, often in the TSL direction. Furthermore, as the internal connection densities of trimer superpatterns increase (i.e., across vertical, black dotted lines in Fig. 7*C*), with the exception of F and C, intramodular utilization also increases, underscoring the importance of signal fine-tuning for local microcircuit interactions.

Next we analyzed the breakdown of superpatterns in the CA1 module as used by pyramidal cells versus interneurons (Fig. 7*D*, blue line). Although many CA1 interneurons interact primarily with pyramidal neurons, many others, including calretinin-positive (CR^+^; Gulyás et al., 1996) and vasoactive intestinal peptide–positive (VIP^+^; Acsády et al., 1996) cells do not, and the interactions of many other interneuron types are unknown. Pyramidal cells dominate the usage of all superpatterns except C, E (a feedforward loop), and K (a double uplinked mutual dyad). Interestingly, in CA1, such diminution is specifically attributable to elevated use of these trimers by perisomatic-targeting (PST) interneurons, namely CA1 axo-axonic cells, horizontal axo-axonic cells, basket cells, basket CCK^+^ cells, and horizontal basket cells (Fig. 7*D*, red dotted line).

### Weighted pattern profile and neuron type fingerprint analysis

**Figure 8.**
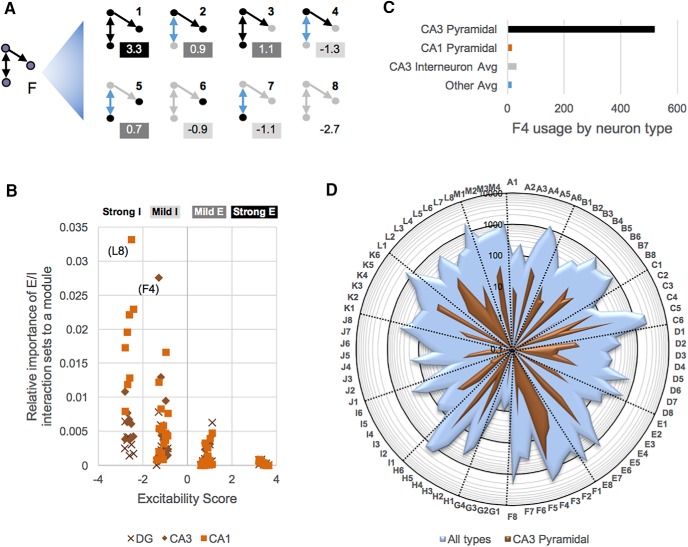
Weighted trimers analysis based on excitatory/inhibitory neuron type distinction. ***A***, The eight patterns that constitute superpattern F, the single uplinked mutual dyad. Black lines and nodes are excitatory, gray lines and nodes are inhibitory, and blue lines indicate reciprocal connections that are excitatory in one direction and inhibitory in the other. ES values are shown in boxes in which background shading indicates strongly and mildly excitatory and inhibitory patterns; key shown in ***B***. ***B***, Relative importance of each ES to the DG, CA3, and CA1 modules. ***C***, Pattern F4 is heavily utilized by CA3 and CA3c pyramidal cells; relatively light usage of this pattern by CA1 pyramidal cells and CA3 interneurons is shown for comparison. ***D***, The CA3 pyramidal cell connected pattern fingerprint (brown) is plotted on top of the overall HC fingerprint (light blue) using a logarithmic scale.

The number of distinct connectivity trimers grows substantially when distinguishing excitatory and inhibitory nodes. After considering rotational symmetry, 104 possible interaction patterns exist between three nodes: 86 fully connected and 18 with at least one disconnected node. For example, eight connectivity patterns correspond to superpattern F, the single uplinked mutual dyad (Fig. 8*A*). The ES captures the overall excitatory or inhibitory nature of a pattern by accounting for the net amplification and dampening of each node by their connected partners. Consider, for instance, pattern F4: a mutually interacting pair of excitatory and inhibitory types, with the former being “uplinked” to a second inhibitory type. Every node in F4 receives a connection from another node and is scored independently. The score of the inhibitory node on the right of the pattern (when rotated as in Fig. 8*A*) is equal to –1.1 because the original value, –1, is amplified (i.e., multiplied) by an inbound excitatory signal. The score of the node at the bottom of the pattern, another inhibitory type that receives excitation, is identical. At the top of the pattern, the original value of this excitatory type (+1) is dampened by an inbound inhibitory edge from the bottom node, yielding a value of 0.9. Summing the scores from all three types gives an overall ES = (–1.1) + (–1.1) + (0.9) = –1.3 for this pattern.

The ES distribution for all 104 trimer patterns has four narrow peaks determined by the number of excitatory or inhibitory nodes therein; accordingly, trimer neuron type patterns may be categorized as strongly inhibitory, mildly inhibitory (e.g., pattern F4, which has two inhibitory types), mildly excitatory, and strongly excitatory. The abundance of inhibitory types over excitatory types in the DG, CA3, and CA1 modules yields higher usages of strongly and mildly inhibitory patterns, consistent with the known diversity of GABAergic interneurons (Fig. 8*B*). Two patterns, L8 and F4, are particularly prominent in certain modules. L8, the all-inhibitory version of superpattern L (Fig. 7*A*) consisting of a feedback loop between two mutual dyads, is used ubiquitously in CA1 to fine-tune inhibition. In contrast, the pervasiveness of F4 in CA3 (and, in fact, the whole HC) is almost entirely due to CA3 pyramidal cells (Fig. 8*C*), which heavily use this pattern to disperse information simultaneously to both CA3 interneurons (many of which supply direct feedback) and CA1 interneurons via the Schaffer collaterals (no direct feedback). In fact, pattern “fingerprint” profiling reveals that F4 is by far the dominant class of interactions for CA3 pyramidal cells (Fig. 8*D*, brown data series; note log radial scale). The overall HC usage of all 86 connected patterns (blue shading) is shown for comparison.

**Figure 9.**
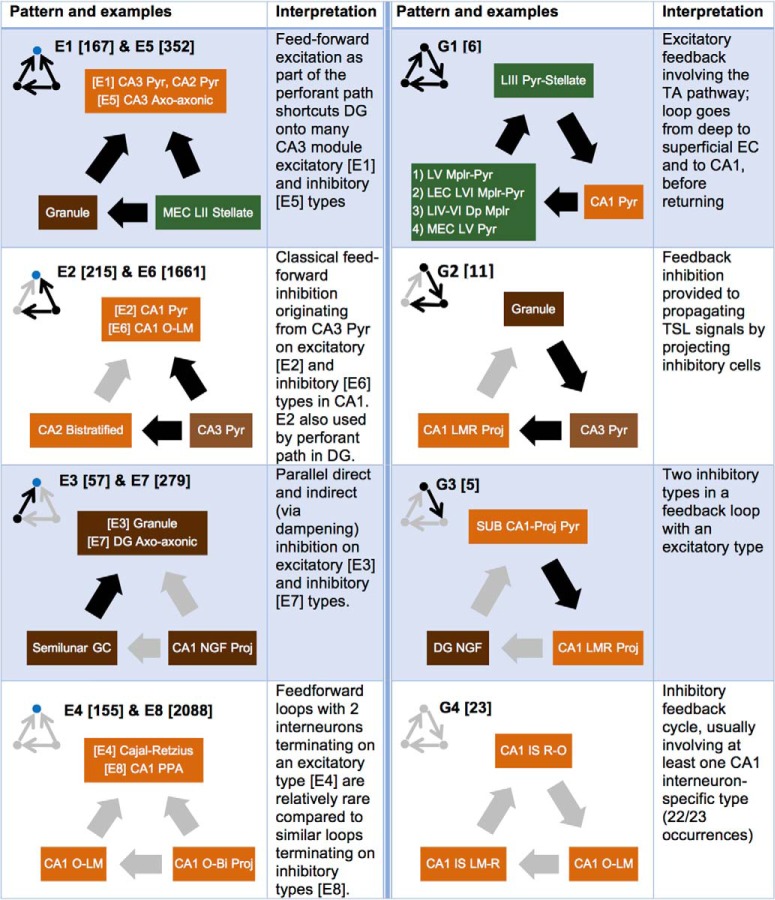
Three-node feedback loops (superpattern G) are generally avoided in HC in favor of other two-step chains, such as feedforward loops (superpattern E). The excitatory/inhibitory combinations of these patterns are displayed in the right and left columns along with representative neuron type groupings and a computational interpretation. Black dots and arrows indicate excitatory types and connections; gray signifies inhibitory types and connections; and blue dots, located at the output of the loop, are excitatory in one pattern combination and inhibitory in the other. Total network occurrences for each pattern are shown in square brackets.

Moreover, HC conspicuously underutilizes superpattern G, along with each of the patterns G1–G4 (45 total interactions of 295,240). Indeed, these patterns, corresponding to a unidirectional feedback loop with no reciprocal connections, are avoided in favor of other structural blocks. When two unidirectional connections transmit signals forward along a chain in HC, nodes 1 and 3 are rarely connected by unidirectional feedback as in G (0.5% of such cases); instead, they are either unconnected in a three-node chain (superpattern B; 38.5%), connected by a feedforward link (superpattern E; 49.8%), or connected by a reciprocal edge that serves both feedback and feedforward purposes (superpattern J; 11.2%). This strikingly uneven distribution is consistent with forward-directional circuitry in which feedback tends to be relatively immediate and curbed (e.g., in the form of a reciprocal edge) or else drawn out over a more global scale. Comparing the counts and circuit locations of feedforward and feedback loops (superpatterns E and G, respectively), along with specific examples and tabulated interpretations, clearly illustrates this trend (Fig. 9). Note that, whereas only four feedback patterns exist due to rotational symmetry, there are eight feedforward patterns.

### Motifs and antimotifs

**Figure 10.**
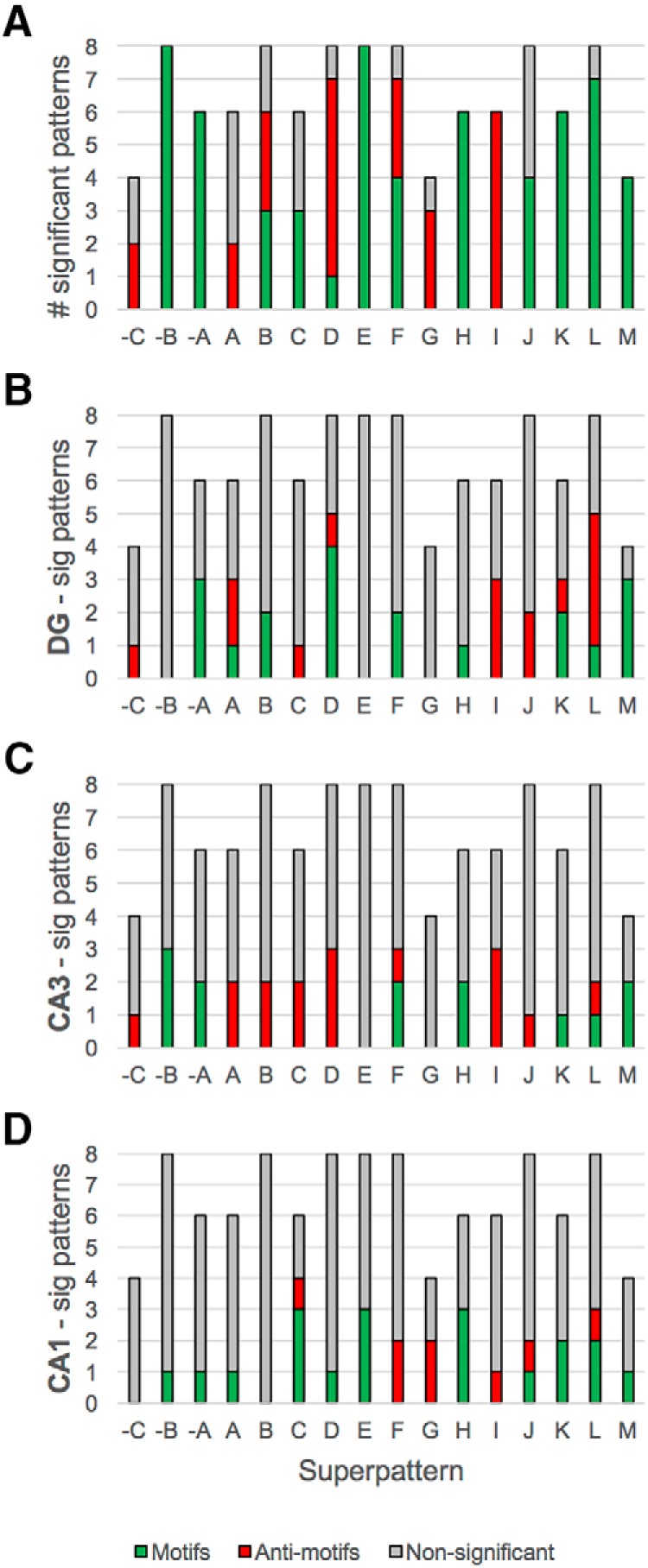
***A***, Motifs and antimotifs are largely determined by HC superpattern topology. Although some superpatterns are neutral, most superpatterns are strong motifs. Only superpatterns D, G, and I are severely underutilized relative to the population of random networks. ***A–D***, The motif/anti-motif balance of individual superpatterns in the network does not necessarily hold for individual modules (e.g., superpatterns C and J).

To identify significantly over- or underrepresented subcircuits, we benchmarked the HC networks to random graphs in which global topology was obliterated, but the underlying composition of all dimers was preserved. Surprisingly, superpattern topology was the most important factor in determining whether a pattern was a motif or an antimotif (Fig. 10*A*). In other words, most superpatterns (13 of 16) are either motifs or antimotifs, independent of the excitatory/inhibitory make-up of their nodes. Only the three-node chain, the single downlink to a mutual dyad, and the single uplinked mutual dyad (superpatterns B, D, and F) contained a mixture of motifs and antimotifs (stacked green and red columns). For the others, the connectivity itself was either over- or underutilized relative to the expectation based on HC dimer distribution. Patterns belonging to D (single downlink to a mutual dyad), G (feedback loop), and I (chain of two mutual dyads) were severely underutilized, but patterns –B (disconnected single edge), –A (disconnected mutual dyad), E (feedforward loop), H (double downlink to a mutual dyad), K (double uplinked mutual dyad), L (feedback with two mutual dyads), and M (fully connected triad) were all strong motifs. These results were robust to PC weighting. Additionally, motifs and antimotifs were module specific (Fig. 10*B–D*). The DG module contained a mixture of motif and antimotifs for many superpatterns. Less densely connected superpatterns, including simple regulation (Alon, 2007), three-node chain, single input module, and single downlink to a mutual dyad (superpatterns A–D) were underutilized in CA3; instead, these superpatterns tended to be slightly overutilized in CA1, where F and G were anti-motifs.

### Pairwise correlations

For the 88 neuron types not located in EC or subiculum, we tested the interactions among 315 connectivity, morphological, molecular, and electrophysiological properties and detected 14,217 (14.3%) significant correlations (*p* < 0.05). These results fell across a spectrum of novelty, and the more interesting outcomes are presented here.

As in the motif analysis, subregional differences in the usage of superpatterns and patterns were revealed. DG types have high participation (relative to other subregions) in three-node chains (superpattern B; *p* < 0.02) but shun dense, highly connected superpatterns, including I (chain of two mutual dyads; *p* < 0.002), J (single point feedforward and feedback loops; *p* < 0.006), K (double uplinked mutual dyad; *p* < 0.00002), L (feedback with two mutual dyads; *p* < 0.00006), and M (fully connected triad; *p* < 0.0005). Interestingly, CA3 and CA1 do not share parallel high or low participation in any superpattern or pattern. Instead, one pattern is highly used in CA1 but avoided in CA3, and one pattern displays the opposite trend. Pattern H6, in which an interneuron acts as a single input module dispersing information to two interneuron recipients with reciprocal feedback between them, is highly utilized in CA1 and underutilized in CA3. Contrarily, pattern L3, a reciprocal edge between two excitatory types, one of which is connected reciprocally with an interneuron and the other of which receives unidirectional information from that interneuron, is avoided in CA1 but highly used in CA3. This connectivity pattern is especially utilized by CA3 and CA3c pyramidal cells to communicate with each other and with a third (interneuron) partner.

Pairwise contingency analysis also detected a set of characteristics differentiating projecting from local neurons. Projecting types participate highly in superpatterns in which information converges to a single point onward through the TSL (*p* < 0.0003): superpattern A (simple regulation) and superpattern E (feedforward loops). Conversely, projecting types use sparingly superpattern C (single input module) and particularly pattern C5 (*p* < 0.0003), which is disperse excitation to two inhibitory nodes. CA1 back-projection cells, a major GABAergic projecting type, are the exception. This neuron type primarily interacts with other hippocampal interneurons and makes use of patterns C6 (inhibitory dispersal to two other GABAergic types) and F8 (Fig. 8*A*) more than any other neuron type.

Last, connectivity was clearly correlated with molecular expression (Hippocampome.org/markers). Although the correlation between subcircuits involving only inhibitory types (e.g., from superpatterns A, D, I, and L) with expression of VIP^+^ and CR^+^ (two markers of interneuron-specific interneurons) was expected, other observations were not. For example, even though somatostatin is not associated with interneuron-specific interneurons, somatostatin-positive (SOM^+^) cell types also tend to interact in groupings with two other GABAergic types (sometimes, but not always, with interneuron-specific types). More specifically, SOM^+^ types are among the top users of the all-inhibitory versions of superpatterns D, E, F, H, J, L, and M. In addition, neurons expressing parvalbumin (including perisomatic-targeting basket and axo-axonic cells) participate copiously in superpattern C (single input module) but sparingly in superpatterns B (three-node chain), D (single downlink to a mutual dyad), J (a single point feedforward and feedback loop), and M (fully connected triad).

### Sensitivity to future additions or subtractions of neuron types

Finally, to examine the robustness of our results to reasonable changes in network size and composition, we reran all analyses on two modified networks. First, we eliminated 26 of the 122 neuron types (asterisks in Table 1), along with their connections, that either were not well known (e.g., described by a single peer-reviewed publication) or contained no or sparse molecular marker and electrophysiological evidence. In the second network, we added 23 new neuron types that are currently being annotated for inclusion in future versions of Hippocampome.org. Remarkably, analysis of both networks yielded results very similar to those reported here for the network of 122 types. Specifically, connection density, CPL, CC, and scaled communication cost were all within 4% of their HC values. More complex analyses (e.g., rich club and motifs) were similarly dependable.

## Discussion

Knowledge about synaptic connectivity between identified neuron types in the hippocampal formation is currently quite scant: empirical information on the presence or absence of synapses is available for less than 1.6% of all possible neuron type pairs. These limited data, however, can be supplemented by leveraging spatially coaligned axonal and dendritic patterns based on the evidence annotated in Hippocampome.org. Although axonal-dendritic colocation does not guarantee synaptic presence, applying the original, neuron-type version of Peters' rule at least reveals the potential connectivity of the full hippocampal circuitry. Although the concept of potential connectivity is extensible in other parts of the brain, it is particularly pertinent in the hippocampus because of its superior structural plasticity (Leuner and Gould, 2010). In particular, in this region, the lack of synapses between neurons at any given moment may not necessarily foreshadow the absence of connection at a different time.

This level of description of the rodent cerebro-hippocampal cortex complements (and fills a gap between) previous large-scale syntheses of tract-tracing studies (Burns and Young, 2000; van Strien et al., 2009) and sparse synaptic sampling (Druckmann et al., 2014). In fact, this effort represents the first comprehensive, literature-based neuron-type circuitry inventory for a mammalian cortical region. Thus, we began to unravel the structural complexity of the hippocampal network through graph theory analyses, shedding light on the functional roles of the component neuron types.

Although networks are quantitatively differentiable according to myriad metrics, two of the most topologically illustrative are CC and CPL (Watts and Strogatz, 1998). We first identified and quantified the specialized topology that brings about higher efficiency and lower overall communication cost in HC than in any equivalent, well-studied network type. Small-world networks, in particular, have been researched and applied fashionably to brain networks for decades (Hilgetag and Goulas, 2016), but we detected significantly higher CC than in equivalent WS networks. The in-built capacity for rapid response times and precise processing, common elsewhere in the brain (Latora and Marchiori, 2001; Bassett and Bullmore, 2006; Rubinov and Sporns, 2010; Mišić et al., 2014), might be especially relevant to the demands of the hippocampus, where the tasks of memory consolidation, retrieval, cognitive navigation, and pathfinding have inherent temporal and spatial constraints.

Next, we exposed a significant modular substructure comprising four densely intraconnected communities. It is worth noting that CA2 and the subiculum, the two hippocampal formation subregions with the fewest known neuron types (five and three, respectively), are currently subsumed into communities dominated by other subregions. CA2 types are split into the CA3 and CA1 modules; subicular types are divided among CA1 and EC. As future knowledge in these areas increases, presumably hailing a proliferation in interneuron diversity, one or both of these subregions might become independent modules.

Regardless, the major high-traffic links between the subregionally based communities recapitulate the TSL and various shortcuts through it. This excitatory relay includes the perforant pathway (PP; grid and border cells from EC layer II to DG and CA3), the temporoammonic pathway (head direction and border cells from EC layer III to CA1), mossy fibers (from DG granule cells to CA3), Schaffer collaterals (from CA3 pyramidal cells to CA1), and the nameless projection from CA1 to EC layer V (Amaral and Lavenex, 2007; van Strien et al., 2009). Although the functional ramifications of these individual conduits are not yet fully understood, in a loop-heavy network that lacks discrete beginning and end points, the detected modules likely act as processing stations regulated by dense intramodular connections (both excitatory and inhibitory). For example, although most studies of the PP focus on the well-known glutamatergic-to-glutamatergic connections onto granule cell dendrites, feedforward inhibition also plays a major role in controlling information processing in DG (Ferrante et al., 2009). As we have shown, the PP can also affect DG interneurons such as MOPP and neurogliaform cells (pattern E2 in Fig. 9). Under physiological conditions, these parallel routes might selectively respond to particular oscillatory input frequencies from EC reflecting different behavioral states (Tateno et al., 2007; Akam and Kullmann, 2010; Ewell and Jones, 2010; Jones and McHugh, 2011). Novelty, for example, induces a slight decrease in granule cell firing rates concomitant with increased DG interneuron activity (Nitz and McNaughton, 2004).

Analysis of OD and ID distributions revealed that the peculiar HC topology was largely due to its axonal architecture, whereas the dendritic circuitry was fairly unremarkable. This result is consistent with the recent finding that the computational load of neurons is unrelated to their ID (Timme et al., 2016); instead, neurons that process large amounts of information tend to receive connections from high-OD neurons. The axonal distribution further pointed to antihub and hub neuron types that utilized highly specific or largely blind targeting, respectively. Hubs are notable because, by directly connecting to many neuron types that are themselves neighbors, they violate typical tenets of wiring minimization (Chklovskii et al., 2002; Chklovskii and Koulakov, 2004; Chen et al., 2006; Bullmore and Sporns, 2012). In fact, both the construction of these superfluous, often long-distance connections and the regular handling of a disproportionately large volume of traffic come at a high cost of energy. At the same time, these nodes facilitate the integration of distributed neural activity and are well situated to integrate the network modules. Such a double-edged nature justifies the “high cost, high value” characterization of these circuit elements (van den Heuvel and Sporns, 2013).

Whereas quantification of modularity yielded informative but nonoverlapping groupings of HC neuron types, the rich club analysis produced a hierarchical ordering of importance of each type to the network. Rich clubs also have been detected in other parts of the brain across several species (van den Heuvel and Sporns, 2011; Harriger et al., 2012; Shanahan et al., 2013; Binicewicz et al., 2015). We identified two nested rich clubs that are likely to route much of the network traffic (Mišić et al., 2014) within the hippocampal formation. Like hubs, this feature accentuates a departure from the parsimonious wiring typically observed in neural systems, but the paths between these critical types provide a highly efficient network core with built-in protection against neurodegeneration. Members of these rich clubs, including the global hubs, are potentially vulnerable to targeted attacks: damaging all neurons within one of these types could lower network efficiency and increase processing times, possibly impairing storage and retrieval functionality. Interestingly, these same types tend to be particularly abundant in terms of cell numerosity, with principal cells present in quantities up to 10 times higher than other neuron types (Bezaire and Soltesz, 2013), thus providing a certain level of resistance against random neurodegeneration. More generally, we showed that the plethora of alternate pathways available in the circuit serves as a second countermeasure.

Finally, we analyzed the superpattern and pattern building blocks responsible for the local interactions that enable global functionality. Three-node subgraphs have attracted considerable attention for their role in complex networks across disciplines (Milo et al., 2002, 2004; Shen-Orr et al., 2002), including neuroscience (Sporns and Kötter, 2004; Song et al., 2005; Santana et al., 2011; Binicewicz et al., 2015). They have been specifically studied in the hippocampus with focus directed at DG mossy cells and unidentified hilar interneurons (Larimer and Strowbridge, 2008) and among recurrent connections of CA3 pyramidal cells (Guzman et al., 2016). Building on this well-defined framework, we added excitatory/inhibitory weights and identified connectivity pattern relations among HC neuron types. In truth, the empirical characterization of even simple (e.g., two-node) interactions between excitatory and inhibitory cells is still vastly incomplete. Recent recordings from more than 500 pyramidal cells and 1500 GABAergic neurons in the mouse neocortex delineated 15 interneuron types that could be grouped based on broad connectivity preferences (Jiang et al., 2015). One group preferentially formed synapses with pyramidal neurons; another, referred to as “master regulators,” connected nonspecifically to all types in proximity of their axons; two additional groups contained interneuron-specific cells that synapsed primarily with other interneurons of the same or of different types, respectively. When these interactions are extended to include a third party (i.e., trimers), the functional implications are more complex. Interneuron-specific cells, in particular, have recently been the subject of much study for their role in disinhibition. Specifically, interneuron-specific cells can influence principal neurons by inhibiting other GABAergic interneurons (Pi et al., 2013; Jiang et al., 2015). In the cortex, this type of circuit control has been linked to enhanced plasticity (Fu et al., 2015) and shown to affect social behavior (Yizhar et al., 2011), sensorimotor integration (Lee et al., 2013), attention (Vogels and Abbott, 2009; Zhang et al., 2014; Sridharan and Knudsen, 2015), and associative learning and memory (Letzkus et al., 2011, 2015). The specific involvement of the hippocampus in many of the above functions makes these connectivity patterns particularly worthy of study.

On a related note, the excitability scores computed for each pattern capture only the overall excitatory/inhibitory nature of a structurally defined trimer. In actuality, each trimer can produce multiple functional states that are affected by the degree of activation, delays in signal propagation, the surrounding neural context, and the behavioral state of the organism (Sporns and Kötter, 2004). Those various functional states of patterns and superpatterns are not analyzed here.

Further meaningful interpretation of our results was hindered by two main factors, both imputable to data incompleteness. First, the neuron types identified in HC are limited to the information available in the literature. Although certain hippocampal areas are well studied (e.g., CA1), other domains, including CA2, subiculum, and entorhinal GABAergic neurons, are still underresearched. Other parts of the subicular complex, including the prosubiculum, presubiculum, postsubiculum, and parasubiculum, are not tracked in version 1.0 of Hippocampome.org. Although morphologically based neuronal-type information was recently reported for the presubiculum (Nassar et al., 2014), on the whole, the breadth of knowledge within these additional areas is relatively narrow. As the scientific community overcomes these shortcomings, the published evidence and Hippocampome.org will grow, which will result in additions and alterations to the connectivity. This accumulating knowledge could affect some of the HC circuit properties described herein. To assess and mitigate this issue, we repeated the analyses on modified (reduced and expanded) networks, and concluded that the main network properties of HC are innate to the well-known, constituent neuron types and unshaken by reasonable additions or deletions.

The second hindrance is the lack of connection weights beyond the binary differentiation of glutamatergic and GABAergic types. A comprehensive solution to this problem requires quantitatively estimating both the counts for each neuron type and the corresponding axonal and dendritic length distributions. Ideally, cell counts would be determined from absolute, stereology-derived numbers for each morphologically defined neuron type, but relative ratios of molecularly defined subpopulations across anatomical parcels could already be useful. These challenging experimental tasks are complicated further by variation across rodent species, strains, ages, sexes, and anatomical axes. Nevertheless, it is generally assumed that principal cells dominate the relative abundances of other neuron types by an order of magnitude (Bezaire and Soltesz, 2013), with experimental observations ranging from 89% of the hippocampal neuron population as a whole (Woodson et al., 1989) to 93% within CA1 (Aika et al., 1994). Accordingly, we also carried out the graph theory analyses with principal cell connections weighted as +10. Remarkably, our conclusions were largely unchanged, and in many cases detailed herein, their significance was even amplified. In addition to obtaining counts for the neuron types, weighting for connections should also be based on measuring the three-dimensional overlap of the neurite trees of each type. However, this approach is currently not feasible, as three-dimensional reconstructions (e.g., from NeuroMorpho.Org) are available for only a small fraction of neuron types. Last, the expression levels of the primary neurotransmitters, as well as the prevalence of membrane receptor proteins in distinct postsynaptic cell types, also play a role in weighting the connections. Although this information, too, is currently lacking, appropriate data can be included in the future to extend potential connectivity analyses beyond binary values.

With the set of tools deployed in this work, future updates to the connectome (through addition, merging, splitting, and weighting of nodes and edges, or through augmentation of known connectivity) can be analyzed in relatively short order. Furthermore, as information accumulates about aging and disease states, the analyses can be repeated with a comparative bent. Extending the foundational results presented here with the expected continuous growth of data will progressively improve our understanding of how network architecture mediates hippocampal function and dysfunction.

## Synthesis

Decisions are customarily a result of the Reviewing Editor and the peer reviewers coming together and discussing their recommendations until a consensus is reached. When revisions are invited, a fact-based synthesis statement explaining their decision and outlining what is needed to prepare a revision will be listed below. The following reviewer(s) agreed to reveal their identity: Gully Burns.

The manuscript describes, a potentially very interesting graph analysis on a connectivity map of the hippocampus and entorhinal cortex, derived from a very significant and well-curated database (Hippocampome.org). As such it is an important paper, that we feel will help the community forward. However, before we can accept the paper, the reviewers request some major restructuring.

1) There is a lack of a clear structure in the manuscript, as there are parts of introduction, materials &amp; methods, legends and discussion mixed up in the Results section; there are new results presented in the discussion (p 27) and the legend texts consist of results and M&amp;M. See examples (pdf) given in the Result section (up to and including section 'Rich club analysis') and legend texts for figures 1 - 5. Color code: Intro = yellow; M&amp;M = green; Results = pink; Discussion = blue; Legend = red. In sum, please write clearly and concisely: his is a scientific report, not a novel.

2) Both reviewers request that the mathematics included in the analysis be described in more detail. The visual output of the various analyses are shown as graphs but it would be very welcome to have a well-defined theoretical narrative in the paper itself (rather than only linking to the available MatLab tools online). A little more detail of the methods would help to introduce these concepts to neuroscience researchers. The inclusion of the reference to Prettejohn et al 2011 helps with this but some local framing of the analysis within the paper would help the reader understand what is being done in each section of the paper.

3) The Discussion is not really a discussion, it is a justification of the use of the Hippocampome data (paragraph 1, p26), a repetition of the results (paragraph 2, p26-27), even the presentation of new results (paragraph 3, p 27). Finally, at the end, two concerns are raised, which both boil down to incompleteness of the database.

4) When the paper transitions between the graph-theoretic model to neuroscience descriptions of circuits there is a small amount of confusion as to whether we're drawing conclusions about cell-types or whole brain regions. (for example, pages 12, 13). The discussion of SOM+ neurons seemed to be concerned with circuits made up of 'three neurons' (meaning what? three neurons from separate cell types?) The authors could review the text to try to make the links between the two types of description a little clearer.

5) In addition, a discussion how existing models of disinhibition might fit into the motif representation and how the logic of other motifs might be explained in terms of the predicted behavior of neuronal populations connected in this way (i.e., neurons inputting cells that send inhibitory signals to an excited pathway). Are there any other criteria to be considered when thinking about these motifs in terms of neural circuits? Some discussion of how the different motifs then might link to model / logical elements in neural circuits might help the reader interpret the constructs being used more easily.

6) Clearly, the number of synaptic weights and the number of cells is an important weighting characteristic that is largely ignored within the existing model (with good reason). The authors discuss +1/-1 weights but clearly there are deeper questions based on the expression of neurotransmitters and receptors in different cell types. Including such information into the underlying representation of the neural circuit, is clearly an important future detail. Would it be possible to include some account of this issue in the discussion?

7) What misses in the Discussion as well, is the interpretation of the presented results in light of what is already reported in the literature (in total there are only 6 references in the Discussion). What are possible explanations for the presented results, what new understandings and possible new hypothesis which can be tested originate from this analysis.

Minor comments

Introduction

5th paragraph: the authors briefly state the rationale of the manuscript " .. how the functionality of the hippocampus relates to its fundamental architectural properties". Unfortunately, this is not followed up in the Discussion.

- the 4th paragraph (about the actual synaptic connectivity) is better suited for the Discussion.

Methods

In general, please provide enough detail about your computations and analysis, such that other scientists could reproduce your experiments.

Section 'a web-accessible ....'

Unable to check this paragraph, keep on getting error messages ... "Unable to launch application"

Section 'Graph theory analyses using Matlab'

- 2nd paragraph: You explain in the first line that CC is the fraction of fully connected triangles. Is a unidirectional connection between the three nodes sufficient or was the threshold set for 3 bidirectional connections?

Results

- The use of '(see methods)' is obsolete (throughout the Result section), because it is standard in scientific papers to write the methods in the Materials &amp; Methods section.

First paragraph of the result (p11 -12)

- This part does not contribute to the results. I would suggest to either put a part of the text in the Method section or use parts to introduce the separate topics in the Results section.

Section 'A highly specialized topic'

- 1st paragraph, line 4: The definition of CC in this section (connected pairs) and in the Methods (fully connected triangles) is not comparable. Please explain.

Section 'Significant communication ... '

- 1st paragraph, line 7: ' ... the red, blue, and purple boxes ...'. These boxes are only explained for DG, CA2 and CA3. The layers of the CA2, Sub and EC are missing. Please add this information.

- 1st paragraph, last two lines about black and white bars: This information belongs to the legend, so please move to legend text of Fig 3.

- 2nd paragraph: The authors discuss 'modules' and 'communities' in the 2nd and 3rd paragraph and in the legends of Fig 3. Are modules and communities identical? Please explain if there is a difference, otherwise use one nomenclature to prevent confusion.

- 2nd paragraph, penultimate line: The authors refer to fig 1B for the modules, but they probably want to refer to Fig 3B.

- 3rd paragraph, first sentence: please provide reference(s) for this statement.

- 3rd paragraph, line 3: Which 4 areas are included in the hippocampal formation? Please prevent confusion, as in the Methods the Hippocampal formation is defined as: "hippocampal formation, defined as the dentate gyrus (DG), CA3, CA2, CA1, subiculum, and entorhinal cortex (EC). " (first sentence of M&amp;M).

- 3rd paragraph, line 4: The authors are referring to Fig 1C. However, Figure 1 only has 2 panels, A and B. Please correct.

Section "Degree distribution ... '

- 1st paragraph, line 5: please provide reference for the statement '...indicating a right-shifted distribution attributable to the presence of network hubs'.

- 1st paragraph, line 8: please provide reference for the statement '...if not thinner-than-normal, tail'.

- 2nd paragraph, line 1 / 2): please define which data subset is used and which analysis is performed. Also include the number of cells.

- 3rd paragraph: In this paragraph the authors discuss hubs. As far as I can see, they have never defined what a hub is. Please add a description of your definition of a hub.

- 4th paragraph, line 9: please describe how you define a provincial hub.

Section "Rich club ..."

2nd paragraph, line 1 / 2: what are flat neuron types?

Section Characteristic connectivity ... '

1st paragraph, line 7: Please explain why disconnected motifs and motifs with 2 nodes are included.

2nd paragraph, line 8: The authors use the word 'remote', please explain what the distance must be to call a neuron remote.

2nd paragraph, line 17 and further: The authors analyzed the motifs found for CA1 pyramidal and interneurons separately. Please explain the reason for this analysis, as it is already known that most interneurons project mostly onto excitatory cell (with the exception of VIP cells).

Section "Weighted pattern ... '

- 1st paragraph, penultimate line: If the excitatory input is valued as 1 and the inhibitory input is valued as -1, how can you yield a final value of 0.9 for the top node? Please explain.

- 1st paragraph, last line: How do you sum up to an ES of -1.3? (we have -1 for the node on the right, a -1 for the bottom node and a 0.9 for the top node).

- 1st paragraph: this part is almost an exact copy of what is written in the M&amp;M. Please remove one of the two.

- 2nd paragraph, line 7 / 8: What do the authors mean with L8? In fig 8A we see F1 - 8, no examples for motif L.

Discussion

In the results the authors state on page 15: "The "CA3 module" included all 25 types from CA3

and four out of five types from less-researched CA2." What does this mean? Can we treat CA2 and CA3 as one structure in the future?

ES values for motifs: you assume that the inputs (E and I) are simultaneously present in all 3 nodes of the motif. How likely do you think it is, that all cell receive input at the exact same time? What is the consequence of this assumption on the conclusions?

How certain can you be that the coexistence of dendrites with an axon terminal in the same parcel, gives rise to a synaptic connection?

Figures

Figure 1 A: Please explain '+',' -' and '.' signs in legend text.

Figure 1 A: What is the meaning of the neurons in bold?

Figure 1B inset: What is the use of this 'toggleable' interactive tool in relation to this paper?

Figure 2A: legends are only provided for 4 out of 8 data points representing the discussed networks. Please consider.

Figure 3C: As far as I could see, there is no reference made in the text to fig 3C.

Figure 4: - Please explain y axis label 'p(k)'.

Figure 5A - top: The y axis label 'Histogram' has no meaning.

Figure 5A - bottom: explain 'd' in legend text.

Figure 5D: very difficult to make sense of this panel. Consider to clarify. What is the purpose of this figure?

Figure 6D: Please describe the color code.

Figure 6E: Please describe the color code.

Figure 8B: What do the authors mean with L8?

Provide a list with abbreviations.
